# Whole-genome analysis of *Mustela erminea* finds that pulsed hybridization impacts evolution at high latitudes

**DOI:** 10.1038/s42003-018-0058-y

**Published:** 2018-05-31

**Authors:** Jocelyn P. Colella, Tianying Lan, Stephan C. Schuster, Sandra L. Talbot, Joseph A. Cook, Charlotte Lindqvist

**Affiliations:** 1 Division of Mammals, Museum of Southwestern Biology and Department of Biology, 1 University of New Mexico, MSC03-2020, Albuquerque, NM 87131 USA; 2 Department of Biological Sciences, University at Buffalo (SUNY), 109 Cooke Hall, Buffalo, NY 14260 USA; 3 School of Biological Sciences, Nanyang Technological University, 60 Nanyang Dr., Singapore, 637551 Singapore; 4 Singapore Centre on Environmental Life Sciences Engineering, Nanyang Technological University, 60 Nanyang Dr, Singapore, 637551 Singapore; 5 U.S. Geological Survey Alaska Science Center, 4210 University Dr., Anchorage, AK 99508 USA

## Abstract

At high latitudes, climatic shifts hypothetically initiate recurrent episodes of divergence by isolating populations in glacial refugia—ice-free regions that enable terrestrial species persistence. Upon glacial recession, populations subsequently expand and often come into contact with other independently diverging populations, resulting in gene flow. To understand how recurrent periods of isolation and contact may have impacted evolution at high latitudes, we investigated introgression dynamics in the stoat (*Mustela erminea*), a Holarctic mammalian carnivore, using whole-genome sequences. We identify two spatio-temporally distinct episodes of introgression coincident with large-scale climatic shifts: contemporary introgression in a mainland contact zone and ancient contact ~200 km south of the contemporary zone, in the archipelagos along North America’s North Pacific Coast. Repeated episodes of gene flow highlight the central role of cyclic climates in structuring high-latitude diversity, through refugial divergence and introgressive hybridization. When introgression is followed by allopatric isolation (e.g., insularization) it may ultimately expedite divergence.

## Abstract (web summary)

Jocelyn Colella et al. report whole-genome sequences of 10 stoats (*Mustela erminea*) from four regions of glacial refugia. They find evidence for two past introgressive events between lineages that coincide with interglacial periods, a pattern that may extend to other high–latitude species.

## Introduction

Glacial refugia are fragmented pockets of unglaciated land where terrestrial species have persisted and diverged through ice ages, and which have significantly structured biological diversity at high latitudes^1–3^. Three primary macro-refugial locations are hypothesized to have enabled the persistence of temperate terrestrial North American species through Pleistocene glacial cycles (>24 glacial/interglacial cycles; 2.6 Mya–11.7 kya). These include an expansive Northern Beringian refugium shared with Asia^4^ and two Southern refugia, separated into East and West by the Rocky Mountains or Great Plains/Mississippi drainage^5,6^. A fourth, smaller refugium (or series of refugia), hypothesized to exist on areas of exposed continental shelf during the last glacial maximum near contemporary North Pacific Coastal archipelagos^7–10^, may have provided another isolated sanctuary for long-term coastal persistence of terrestrial species. Although this refugium has received varying degrees of empirical support, its existence is critical to understanding dynamics of intercontinental biotic exchange and it may have played a primary role in the early peopling of the Americas^11^.

Phylogeography of high-latitude *Mustela erminea*, the stoat or ermine (Family: Mustelidae) delineates four genetically distinct mitochondrial lineages geographically corresponding to these four refugia^12^. Although glacial isolation appears to have impacted the long-term evolution of this mammalian meso-carnivore, the effects of glacial recession and subsequent secondary contact between refugial lineages remain unexplored. Therefore, we investigated the consequences of cyclic climates and pulsed introgression on genomic diversity in this circumboreal species complex. During inter-glacial warming, divergent lineages expanded from refugial centers until they reached environmental or biological barriers^13^. In some cases, admixture occurred upon secondary contact with other lineages. If *M. erminea* experienced serial bouts of climate-mediated introgression, a repeated signature of introgressive hybridization should be evident in the genomes of refugial descendants^14^.

Contact zones are windows into evolutionary processes^15^ now being explored on a genomic scale. We define introgressive hybridization as interbreeding and the movement of alleles between two genetically distinct lineages. Although often difficult to parse introgression from incomplete lineage sorting in closely related taxa or lineages^16^, investigations of divergent lineages, such as those within the *M. erminea* complex, provide insight into the genomic consequences of introgression. For example, genetic swamping or homogenization can prevent divergence between mixing lineages, while Bateson–Dobzhansky–Muller^17–19^ and mitonuclear^20^ incompatibilities can lead to hybrid breakdown, thereby promoting divergence through reinforcement^21,22^. Although our understanding of hybridization emerges largely from research on plants^23,24^, the genomic era has uncovered substantial reticulate evolution (e.g., introgressive hybridization) in a growing number of animal species^25,26^, including humans^27^. As histories of divergence and speciation-with-gene flow continue to be discovered (e.g.,^28,29^), the implications of hybridization for the conservation of evolutionarily distinct units appear profound^30^.

To test whether genomic structure in stoats reflects refugial origins and to explore the consequences of contact among lineages, we generated ten whole-genome sequences of representatives from each refugial clade. With expanded genomic coverage, we test for signatures of refugial divergence and introgression, characterize the timing of introgression events relative to climatic oscillations, and infer the impact of introgressive hybridization on divergence across the complex landscape of northwestern North America.

## Results

### Refugial origins and phylogeography

**Fig. 1:**
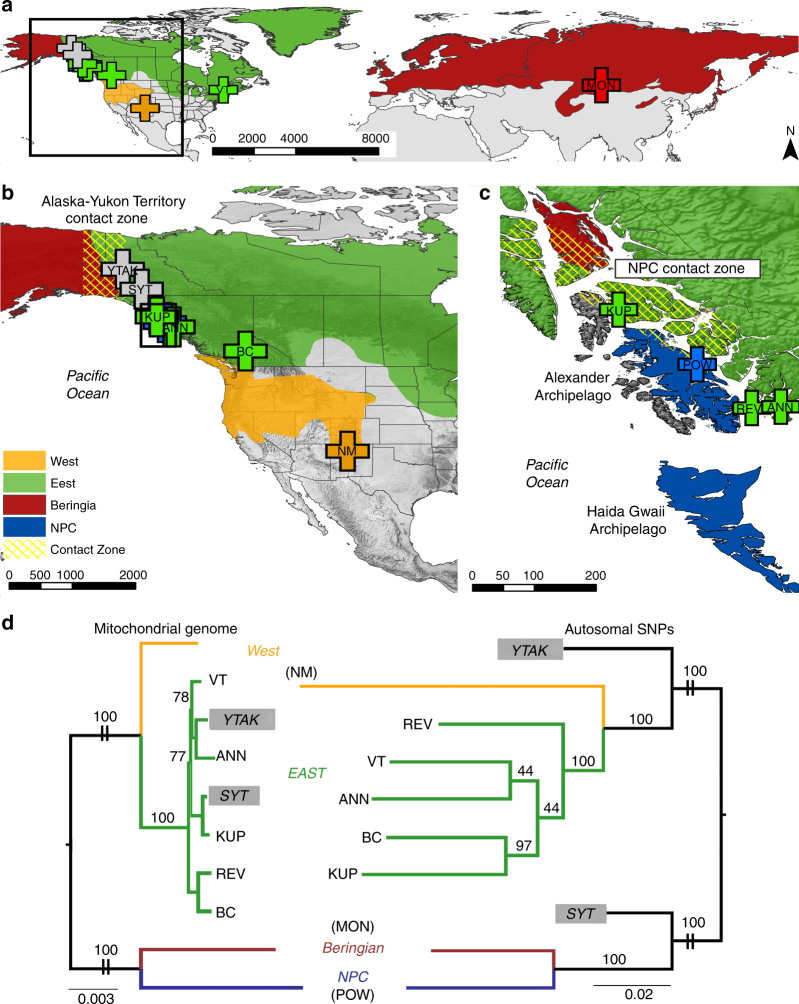
*Mustela erminea* clade geographic distributions and phylogenetic relationships. **a**–**c** Stoat clade distributions (clade names reflect refugial origins) based on amplicon mitochondrial haplotype distributions^12^ and IUCN (International Union for Conservation of Nature, www.iucn.org) range information. Genomic samples are labeled with locality abbreviations: ANN (Annette Island, Alaska, USA), BC (southern British Columbia, Canada), KUP (Kupreanof Island, Alaska, USA), MON (Mongolia), NM (New Mexico, USA), POW (Prince of Wales Island, Alaska, USA), REV (Revillagigedo Island, Alaska, USA), SYT (Southern Yukon Territory, Canada), YTAK (northern Yukon-Alaska border), VT (Vermont, USA). Contact zones are denoted with yellow hatching, based on the presence of mixed mitochondrial haplotypes^12^. Scale bars are in kilometers. **a** Global sampling scheme. **b** North American sampling localities. **c** Sampling within the northern North Pacific Coast (NPC) archipelagos. **d** Mid-point rooted maximum-likelihood phylogenies, scaled by genetic distance. **d** (left) Complete mitochondrial genomes and **d** (right) autosomal SNPs for ten *M. erminea* individuals. Phylogenies demonstrate strong support for four major refugial clades and mitonuclear discordance for hybrid samples (SYT, YTAK). Deep divergence between the NPC Island (POW) and Beringia (MON) clades is evident in the long branches in both phylogenies

To test our hypothesis that the four mitochondrial stoat lineages originated as the result of isolation and divergence in each of the four major North American refugia, we generated whole-genome sequences for ten *M. erminea* drawn from each refugial lineage and two spatially disjunct contact zones (the interior border of Alaska-Yukon Territory and the NPC; Fig. 1). We contrasted mitochondrial and nuclear phylogenies against geographic clade distributions, estimated pairwise diversity metrics (pairwise mitochondrial and nuclear divergence, *F*_ST_^31^, relatedness^32^), and used ADMIXTURE^33^ analyses to define populations.

**Fig. 2:**
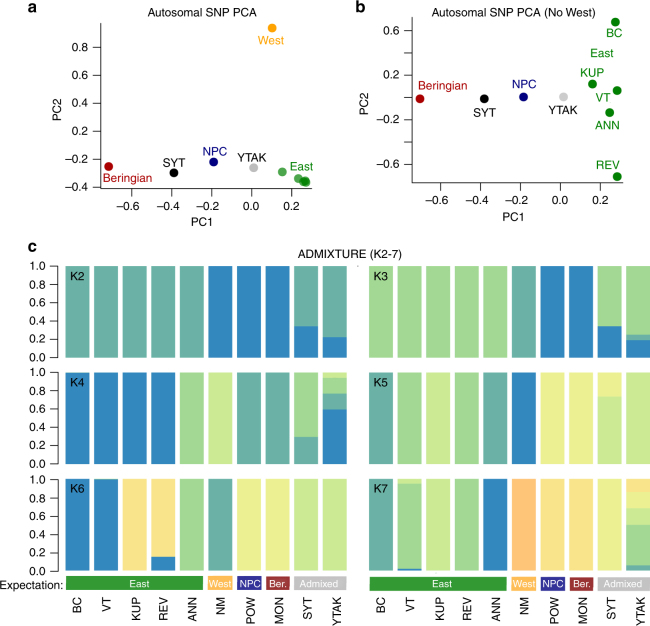
Principal component analysis and ADMIXTURE results. **a**, **b** SNP principal component analysis (PCA). All samples identified as admixed by *f3*-statistics (NPC, SYT, YTAK) lie intermediate to Beringia and East clades, their purported source populations. **a** PCA with all samples demonstrates substantial nuclear genetic differentiation of the West clade. PC1 explains 20.7% of the total variation, and PC2 explains 14.1%. **b** PCA with the West population removed demonstrates additional structure within the East clade, centered on Vermont (VT). Hybrid samples are intermediate to their source populations. PC1 explains 29.8% of the total variation and PC2 11.8%. **c** ADMIXTURE plots for K2–7. K = 3 best matches our expectation based on the geographic distributions of refugial clades and is consistent with admixed ancestry for SYT and YTAK samples: (1) East samples as a distinct group, (2) a West population, and (3) a combined Beringia-NPC population with two hybrids admixed from these groups. K = 6 has the lowest cross-validation score for our data and shows an identical pattern to K3 but with additional substructure within the East clade, indicative of microrefugial substructure near Appalachia and consistent with PCA results suggesting East expansion from a single refugium

**Fig. 3:**
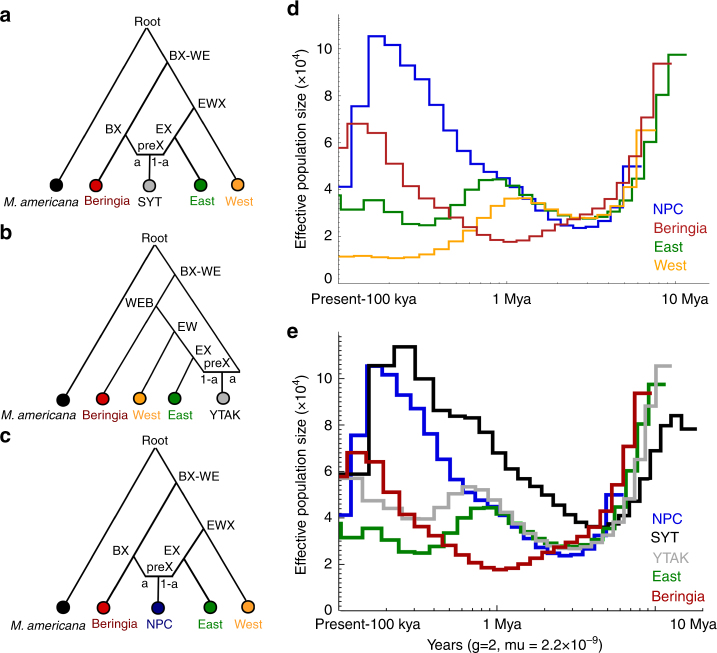
Phylogenetic placement of admixed samples and historical demography. **a**–**c** Optimal phylogenetic placement of hybrid samples onto our non-admixed RAxML topology (see also Supplementary Fig. 5) based on *f4*-statistics fitting in Admixture Graph. **a** SYT originates from ancestors of Beringia and East populations; **b** YTAK is admixed from East and an unsampled ancestor of all *M. erminea* lineages; **c** NPC stems from ancestors of Beringia and East clades. (**d** distributions of effective population sizes (*N*_e_) through time from pairwise sequential Markovian coalescent (PSMC) analysis scaled by a general mammalian mutation rate (2.2 × 10^−9^) and a 2-year generation time. **d** PSMC plots for a representative from each refugial population (East, West, Beringia, NPC). **e** PSMC plots for hybrid samples (YTAK, SYT, NPC) and their source populations (Beringia, East). Hybrid distributions mimic that of their most-backcrossed source *(f*_*4*_*)* with artefactual *N*_e_ inflation, as expected for admixed demographic histories

Our results showed strong mitochondrial (Fig. 1; Supplementary Fig. 1) and nuclear (Figs. 1–3; Supplementary Fig. 2; Supplementary Table 1) support for four stoat clades geographically coincident with North American refugia. We identified deep divergence of a highly distinctive West clade, a group previously lacking strong nuclear support^12^ (Figs. 1, 2a and c; Supplementary Fig. 2; Supplementary Tables 1 and 2). We detected substantial genetic divergence between mainland stoat lineages (e.g., 21% average pairwise difference across >3 million autosomal SNPs between Beringia and East clades), comparable to estimates between Beringia stoats and the outgroup species, *M. putorius* (domestic ferret, 20%), calling into question whether this widespread mustelid is a single species (Supplementary Table 1). Centrality of the Vermont sample is consistent with widespread expansion of the East lineage from a single eastern refugium followed by intra-clade divergence (Fig. 2b). Similar refugial signatures have been detected in other North American species (e.g., martens^34^ and black bears^35^). Additional structure within the East clade (Fig. 2b) may reflect microrefugial diversification^36^. Geographic proximity (Fig. 1) is not predictive of genetic similarity (Fig. 2b; e.g., REV and BC), consistent with patterns also uncovered in primary stoat prey species such as voles^37^ and lemmings^38^.

**Table 1:** Source populations and admixture date estimates for each hybrid sample

Target	Source1	Source2	*f3*-statistics			MixMapper date estimates		
			*f3*	Err	*Z*	*α*	Mixed drift	Years (*N*_e_ = 375k)
NPC	East	Beringian	−0.05	0.009	−5.28	0.48–0.59	0.00–0.00	0
SYT	East	Beringian	−0.17	0.003	−48.66	0.52–0.78	0.00–0.00	0
YTAK	East	Beringian	−0.15	0.003	−47.41	0.48-0.73	0.00–0.23	0–393,996.5

Admixture date estimates in years based on an effective population size (*N*_e_) of 375k (ref. ^12^) for the North Pacific Coast island (NPC) and the two Alaska-Yukon hybrids (SYT from Southern Yukon and YTAK from the northern Alaska-Yukon border). Err indicates the standard error and *Z* indicates the *Z*-score

The sister relationship between NPC and Beringia stoats, combined with substantial genetic divergence (nuclear >10%; mitochondrial >4%; Supplementary Table 1) between these clades and their current geographic distributions, are consistent with the Coastal Refugium Hypothesis^7–10^. Ancestry estimation^33^ identified autonomous East and West populations but a single combined Beringia/NPC population (K = 3; Fig. 2c), again highlighting the paleoendemic ancestry of the NPC Island lineage. Pairwise mitochondrial divergence is also particularly elevated for the NPC Island lineage, bordering the species-level divergence threshold suggested for distinct mammal species^39^.

### Detecting introgression and determining source populations

Evidence of recurrent introgressive hybridization, whether as a single or recurrent event, should be evident in the genomes of refugial descendants. We hypothesized that signatures of introgression should be present in individuals collected within the Alaska-Yukon Border hybrid zone and expected that stoats from Beringia and East clades are the most likely source populations based on contemporary clade distributions. In addition to contrasting mitochondrial and nuclear phylogenies for discordance, we performed a series of tests for introgression including ADMIXTURE^33^ and *f3*-statistics (Supplementary Table 3)^40,41^ to assess shared genetic drift among all possible combinations of two source populations^42^.

We identified mitonuclear discordance for two individuals collected near the Alaska-Yukon border (herein SYT and YTAK, representing Southern and Northern localities along the contact zone, respectively), which can indicate a history of genomic introgression or incomplete lineage sorting. ADMIXTURE identified the same two individuals as admixed between East (K3: SYT 66%, YTAK 74%) and Beringia (K3: SYT 34%, YTAK 19%) clades or as a single, but distinct population, consistent with shared source populations (Fig. 2c). Both Alaska-Yukon samples were positioned between their hypothesized source populations in our PCA results (Fig. 2a and b), with SYT closer to Beringia and YTAK closer to the East clade, further supporting their mixed ancestry and suggested asymmetric backcrossing distributions. Lastly, *f2* statistics, a measure of distance between populations, were also lowest between the putative Alaska-Yukon hybrids and their suspected source populations, further supporting their derivation from the same source populations (Supplementary Table 4).

Surprisingly, NPC Island stoats also tested positive for admixture, with Beringia-East parentage (Table 1; Fig. 2a and b). These island stoats have been isolated off the NPC (Prince of Wales Island and the neighboring Haida Gwaii archipelago of British Columbia, Canada [not sequenced here]) for at least 10,000 years^12^; therefore, we suspect they are derived from an ancient hybridization event that occurred during an earlier interglacial period, prior to the contemporary insularization of this clade. We found no evidence of admixture, however, in the Kupreanof Island sample.

### Testing for asymmetric introgression

Biased or asymmetric introgression may indicate a selective advantage, the existence of genetic incompatibilities, or a demographic imbalance. Alternatively, equal contributions from both parental source populations may suggest that there are minimal genetic incompatibilities between hybridizing stoat clades, as we would expect within a single species. For the NPC Island clade, biased allelic overlap with one source population may shed light on interglacial colonization dynamics, as the island may have been colonized by both Beringia and East ancestors who subsequently interbred. We used *F4-*statistics, similar to *D*-statistics^43,44^ and ABBA/BABA^45^, to highlight differential backcrossing histories among lineages and used admixture graph fitting^46^ to estimate the topological placement of admixed samples relative to their potential sources.

First, our *f4* results highlight the influence of repeated glaciation in North America as reflected in multiple episodes of gene flow between lineages originating in independent refugia. Allele sharing (*f4*) reflected phylogenetic relationships, with stepwise genetic distance from the ancestral Beringia clade increasing with greater geographic distance from Beringia (Fig. 1; Supplementary Tables 5 and 6), a pattern consistent with more frequent or prolonged contact between geographically proximal refugial lineages.

Admixture graph fitting identified SYT as the result of Beringia and East admixture, while YTAK was produced from an ancestral admixture event between East and a shared ancestor of all stoat lineages (Fig. 3a and b; Supplementary Table 7). The vast majority of *f4* comparisons (Supplementary Tables 5 and 6) were highly significant (*Z*-score >5), reflecting substantial intra-specific divergence within *M. erminea*. Biased allelic overlap suggested that YTAK shares more alleles with the East source, whereas SYT is more backcrossed with the Beringia source (Table 2). Differentially backcrossed hybrid offspring within this broad, mainland contact zone suggests that hybridization between East and Beringia lineages may have minimal negative fitness consequences (e.g., reduced fertility, viability)^47^. These preliminary inferences require additional sequencing of hybrids from the interior Alaska-Yukon hybrid zone to characterize the specific genomic regions that may be crossing semi-permeable lineage boundaries and to rigorously test for asymmetric introgression, which could be driven by selection (e.g, genetic incompatibilities, Darwin’s corollary to Haldane’s rule^48^), drift, or demographic parameters. If gene flow is neutral, extended periods of contact may promote fusion between East and Beringian lineages. However, the contemporary persistence of both Old World Beringian and New World East lineages in North America suggests that gene flow during brief periods of interglacial contact through the Pleistocene were insufficient to homogenize refugial divergence.

**Table 2:** *f4*-statistics for hybrid samples (*X*) with *M. putorius* outgroup (*W*)

*W*	*X*	*Y*	*Z*	*f4*	*Z*-scores
OG	NPC	East	Beringia	0.30	31.79
OG	NPC	West	East	0.32	76.99
OG	NPC	West	Beringia	0.51	70.42
OG	SYT	East	Beringia	0.13	9.29
OG	SYT	West	East	0.37	86.48
OG	SYT	West	Beringia	0.41	42.17
OG	YTAK	Beringia	East	0.28	27.12
OG	YTAK	West	East	0.40	90.67
OG	YTAK	West	Beringia	0.08	10.18

*F4*(W,X:Y,Z) results for each hybrid sample (*NPC* North Pacific Coast island, *SYT* Southern Alaska-Yukon border, and *YTAK* northern Alaska-Yukon border) relative to all possible parental source populations (East, West, Beringia)

All *Z*-scores are positive for ease of interpretation and significant (>5). Rotating the order of a pair (e.g., YZ to ZY) switches the sign +/− of both the *f4*-statistic and the *Z*-score but does not change the relationships

Biased allele sharing between NPC and Beringia (Table 2) is consistent with phylogenetic relationships (Fig. 1d) and three possible evolutionary histories: (1) differential introgression (selection) favoring Beringia alleles early in the NPC Island clade’s origin; (2) extended or more frequent connection between Beringia and NPC refugia throughout the Pleistocene; or (3) stochasticity, potentially compounded by founder effects or bottlenecks.

### Timing introgressive events

Given the pattern of North American glaciation^49^, the current geographic isolation of the NPC Island clade, and the limited dispersal abilities of insular *M. erminea*^50^, we suspect that hybridization is not ongoing on NPC islands. In contrast, based on the current distribution of mitochondrial diversity^12^ and our understanding of postglacial population expansion, we expect that stoats from East and Beringia clades are actively interbreeding along the Alaska-Yukon border. To approximate the timing of admixture events, we converted drift unit branch lengths (*D*), output from MixMapper^51^, to absolute time (years) using the formula *D* ≈ 1−*e*^−^^*t*/2*N*e^(solved for *t* generations)^52^.

The estimated time of admixture for Alaska-Yukon hybrids did not differ from zero (Table 1), suggesting that the Alaska-Yukon Territory border is an active hybrid zone between the leading-edge of Beringia and East stoat clades. In contrast, NPC admixture is estimated at up to 394 kya (Table 1; Supplementary Table 8), prior to the Wisconsin glacial (30–80kya^53,54^) and Sangamonian interglacial (80–140kya^53,54^). The weaker NPC hybrid signal relative to contemporary Alaska-Yukon hybrids (*f3* *Z*-score), the insular isolation of this clade for at least the past 10,000 years^12,55^, and the short generation time of stoats (2 years^56^) suggest that NPC Island admixture is more ancient than that occurring in the mainland Alaska-Yukon zone. We hypothesize that the hybrid origin of the NPC clade predated a period of glaciation and refugial isolation, but this point requires further exploration with expanded sampling and tests of linkage disequilibrium. If NPC stoats continue to evolve in isolation, our results suggest that continued divergence in allopatry may ultimately lead to speciation.

Based on our results, NPC islands were colonized either by admixed individuals or independent invasions of both parental lineages (East, Beringia) that subsequently hybridized. Introgressive hybridization, preceding divergence in isolation, may have disproportionately elevated the evolutionary distance between NPC Island stoats and other lineages (Figs. 1, 2, and 3d, e; Supplementary Tables 1–4). Isolation of a genetically recombinant population, at least through the Last Glacial Maximum, in a coastal refugium reinforced an evolutionary trajectory independent from either source population (Supplementary Fig. 3). Further, low amplicon variation in NPC stoats^12^ suggests that island founder effects or a bottleneck^5^ occurred among NPC stoats in the coastal refugium^57^. Drift may impact small populations and may have further accelerated NPC stoat differentiation.

### Historical demography

We expected glacial cycling (periods of isolation/contact) to have impacted the historical demography of each refugial stoat clade differentially. Further, we hypothesized that individuals with a history of introgression will exhibit elevated effective population sizes (*N*_e_), with a distribution most similar to that of their most-backcrossed source population (East for YTAK, and Beringia for SYT). Last, we expected cladogenesis to coincide with refugial isolation during glacial periods and demographic troughs, while *N*_e_ is expected to rise during interglacials as a consequence of population expansion.

Stoat demographic histories (PSMC^58^; Fig. 3d, e) showed clade-level responses to cyclic glaciation, highlighting the central role of large-scale climatic shifts in shaping diversity at high latitudes through episodic isolation and contact. Overall low, but relatively stable *N*_e_ in East and West clades reflected similar demographic responses to New World glacial cycling, where the West refugium was likely smaller than the East, as reflected in smaller *N*_e_ (Fig. 3d). Unlike Nearctic populations, Western Beringia experienced substantial expansion historically, corresponding to reduced glaciation in eastern Eurasia and the larger size of Beringia compared to the NPC refugium. Dates of cladogenesis mirrored estimates from the calibrated mitochondrial genome phylogeny (Supplementary Fig. 1), with inter-clade divergence beginning around 2 Mya when Beringia split from the other stoat lineages, followed by West (1.3 Mya) divergence, and most recently Island/East (0.9 Mya) cladogenesis (Fig. 3d). However, the hybrid ancestry of the NPC island sample suggest that cladogenesis estimates based on *N*_e_ may be unreliable. As expected, when hybrid distributions are juxtaposed against their parental populations (Fig. 3e), they mirror that of their most-backcrossed source population, but with an upward shifted *N*_e_^59^. If the increase in hybrid *N*_e_ occurs at the time of hybridization between parental populations^59^, SYT and YTAK admixture may be older than predicted based on admixture date estimates alone.

Combined with diversity metrics (Supplementary Fig. 2; Supplementary Tables 1–4), highly distinct demographic histories suggest taxonomic revision of *M. erminea* may be warranted, pending wider geographic sampling. This is a particularly compelling example of cryptic diversification in carnivores when contrasted against weakly differentiated PSMC demographic histories uncovered in some canid^60^ (wolf, dingo, domestic dog) and felid^61^ (tiger, leopard, cheetah) genomes.

## Discussion

We document multiple temporally and spatially offset bouts of introgressive contact between divergent stoat lineages, corresponding to distinct phases of glacial cycling. Therefore, we predict that other higher latitude species with shared biogeographic histories may also exhibit genomic signatures of repeated introgression corresponding to cyclic climates. This process of recurrent introgressive hybridization between geographically proximal lineages, followed by refugial or insular isolation and divergence, may contribute to elevated levels of island endemism, particularly for continental archipelagos adjacent to glacially dynamic mainland areas.

When backcrossing occurs into both parental populations, as in the Alaska-Yukon hybrid zone, introgression may be stochastic or selectively neutral. Consequently, extended periods of contact may lead to homogenization^18,62^ and result in a net loss of regional diversity^63^, such that the divergent lineages evident today (East, Beringia) may be ephemeral^64^. The narrow Alaska-Yukon hybrid zone and strong East-Beringia genomic differentiation, however, suggests that such peripheral hybrid populations are ephemeral and minimally contribute to long-term evolution of high latitude diversity. If introgression events are temporary, genomic evidence of serial hybridization offers a model for detecting refugia and tracking colonization routes among high latitude species. Here, we provide yet another example of introgressive hybridization in mammals, highlighting both the prevalence and varied role of introgression in mammalian evolution. Our results demonstrate that, for geographically widespread species, the long-term evolutionary role of introgression often may be inconsequential relative to larger biogeographical processes, such as island vicariance, refugial isolation, or rapid environmental change.

In some situations, however, a population bottleneck in a small refugium or in response to insularization can enable the temporary persistence of less fit recombinant genotypes^65^. Even a temporary reduction in fitness can enable traversal between fitness-landscape optima and promulgate alternative evolutionary trajectories, which can permanently impact the genomic structure of high latitude populations and potentially lead to speciation^65,66^. Genomic evidence of hybridization, followed by divergence in geographic isolation, is consistent with a hybrid origin for the NPC lineage. Though still incipient in this case, the process of introgression followed by allopatry may lead to hybrid speciation^67^. Hybridization can catalyze the evolution of reproductive barriers^68^ or expedite divergence through the rapid generation of novel genetic recombinants. Although the mechanisms underlying this special case of divergence with gene flow remain elusive, a plausible precondition for speciation is the separation of lineages into allopatric ranges, which may enhance the genetic divergence of a recombinant population, even if introgression is not directly implicated in speciation. Therefore, NPC stoats potentially highlight the integral role of post-hybridization allopatric divergence in the evolutionary histories of island and refugial taxa, but expanded insular sampling is required to fully test this mechanism of divergence.

It has long been argued that pre-zygotic isolation is reinforced when diverging groups return to parapatry and hybridize^18,20^, although this idea is not without controversy. Under this scenario, hybridization between incipient species can result in unfit hybrids and natural selection might act against hybridization, reinforcing pre-zygotic isolation mechanisms, eventually leading to speciation and avoiding maladaptive hybridization^18,69^. In this fashion, continuing and progressive reinforcement of pre-zygotic mechanisms would ultimately result in speciation and complete isolation between lineages^18,70^, if hybridization and selective pressures on both lineages are symmetrical^71^. With an active and plausibly ongoing hybrid zone, along with more ancient island admixture, stoats provide a unique opportunity to investigate the impact of symmetrical versus asymmetrical introgression using genomics.

With multiple waves of mainland colonizers corresponding to climatic cycles and potentially adding novel genetic elements to insular genomic architectures, the process of repeated, climate-mediated hybridization may have played a role in diversification across other land-bridge island systems. Irish stoats, for example, exhibit elevated genetic diversity compared to neighboring populations in Great Britain, despite persisting on a smaller island farther from a continental source^13,72^. In this parallel system, the English Channel separating Britain from the European mainland has been serially exposed through Pleistocene glacial cycles^73^, effectively homogenizing populations across the Channel. On the other hand, the Irish Sea, nearly twice the depth of the English Channel, further separates Ireland from Britain, presenting a formidable barrier to gene flow that allowed only occasional influx of novel alleles from mainland or British immigrants during extreme glacial events. This mechanism of post-hybridization allopatric divergence is not restricted to land bridge or continental archipelagos. Oceanic island populations of Darwin’s storied finches on the Galapagos Islands also were impacted by climate-mediated pulsed hybridization that was coincident with changes in sea level due to glaciation^74^.

Admixture in this insular endemic (NPC Island stoats, subspecies: *M. e. haidarum*) poses a particular challenge for the U.S. Endangered Species Act, which currently denies protection for hybrid taxa^30,75^. *Mustela erminea haidarum* is federally protected in Canada on the Haida Gwaii Islands of British Columbia^56^, but also occurs on Prince of Wales Island, Alaska, where it has no protection. This study is congruent with previous work noting that this island-restricted stoat subspecies is genetically^12,76^ and morphologically^77^ distinctive, but levels of management protection at the federal level differ profoundly between Canada and the United States.

Genomic signatures of repeated admixture and refugial isolation among North American stoats highlight the episodic role of Late Quaternary climate cycles in generating and structuring high latitude diversity. We demonstrate two instances of gene flow between stoat lineages coincident with interglacial periods: one contemporaneous along the interior Alaska-Yukon border and a second, more ancient event, occurring along the NPC that left a signature that persisted through multiple glacial cycles. Each hybridization event exemplifies an antagonistic outcome of hybridization (e.g., homogenization versus diversity generation) within a single species complex, highlighting the multifaceted role of introgression in mammalian evolution and the varied temporal and geographic scales at which this process unfolds. At high latitudes, where dramatic range shifts in response to glacial cycling afforded recurring opportunities for contact and isolation, ephemeral hybridization has repeatedly shaped biological diversity^2,78,79^.

## Methods

### Sequencing, assembly and post-processing

Contact zones and populations were defined based on expanded mitochondrial sampling^12^. Ten whole-genome sequences for representatives from each refugial stoat clade, and with additional sampling in suspected contact zones (Fig. 1), were generated on an Illumina HiSeq 2000 (paired-end reads [100 bp]), three samples in four Illumina lanes and the remaining samples in one lane each (Supplementary Table 9). Liver subsamples were obtained post-mortem from the University of New Mexico’s Museum of Southwestern Biology in compliance with ethical regulations (IACUC #16-200526-MC). We used a DNeasy Blood and Tissue Kit (QIAGEN, USA) extraction. Next-generation sequencing libraries were prepared using the Illumina TruSeq DNA Sample Prep Kit. Our genome assembly pipeline followed Lan et al.^79^ with reads examined using FastQC^80^ and adapter sequences and sex chromosomes removed (Trimmomatic v0.33 (ref. ^81^)). Quality scales were compared across samples and rescaled to Illumina 1.8 + phred + 33 quality score, when necessary, to maintain consistency (Picard v1.9: http://picard.sourceforge.net; SAMTools^82^). Reads were mapped to the domestic ferret genome^83^ (*Mustela putorius furo*) using the Burrows-Wheeler aligner^84^ (BWA). An additional BWA iteration extracted mitochondrial genomes. Final depth of coverage ranged from 10 to 61× (Supplementary Table 9). PCR duplicates were removed using the MarkDuplicates tools from the Picard suite. Nuclear and mitochondrial genome consensus sequences were called using mpileup in SAMtools. SNPs were called using HaplotypeCaller in the Genomic Analysis Toolkit^85^ (GATK) for all stoats and again against each of two outgroups (*M. putorius* and *Martes americana*) and filtered by minimum depth (minDP = 2), genotype quality (minGQ = 30), minimum minor allele frequency (MAF = 0.1), and scaffold size and location (1 Mb), and then private alleles and indels were removed using VCFtools^86^ (Supplementary Table 10). We intentionally selected an MAF of 0.1 to remove singletons (e.g., individual-specific, rare mutations), and thereby any potential sequencing errors, which are more common in low-coverage genomes. Although singletons can be informative when analyzing within population variation, our analyses focus on shared alleles (e.g., *f*-statistics) to identify introgression and backcrossing histories, therefore singletons, since they are not shared, do not provide information about allelic overlap among populations. Our minDP (2) was set as one-third of the coverage of our lowest coverage samples (e.g., roughly one-third of 8×) as recommended by the PSMC manual. Also, 2 is the minimum requirement for detecting heterozygotes. Format conversions (vcf, ped, bed) were conducted using PLINK^87^. Missing data were removed (--max-missing) based on analysis specifications. SNPs were spaced (1 per 50 bp window) to account for linkage disequilibrium (e.g., 25) and sorted into 46 ‘pseudo-chromosomes’ to enable the application of human-specific analyses on a non-model system with 40 chromosomes.

### Phylogenetic methods

jModeltest^88,89^ estimated the most appropriate model of evolution for mitochondrial genomes and autosomal SNPs, with and without outgroup sequences (*M. putorius* and *Martes americana*). We generated phylogenies for the entire mitochondrial genome (calibrated/uncalibrated) and autosomal nuclear SNPs (uncalibrated) using RAxML^90^ (GTRCAT model, 10,000 generations, random starting seed) and Beast2^91^ (1 million generations, 4 chains, 2 runs, sampling frequency of 1000). It should be noted that phylogeny inference using highly variable data (e.g., SNPs) can induce acquisition bias resulting in longer branch lengths^92^, however, other divergence metrics (e.g., PCA, relatedness, *F*_ST_) provide additional evidence of substantial divergence between refugial stoat clades. The oldest known *M. erminea* fossil^93^ (1.8 My) was used as a log-normal fossil calibration (mean = 5.16, sd = 0.25, offset = 180) to estimate divergence times in Beast2 (Supplementary Fig. 1) following the TreeThinkers tutorial (http://treethinkers.org/tutorials/divergence-time-estimation-using-beast/). A log-normal distribution places the highest probability on ages that are somewhat older than the fossil date, with non-zero probability to infinity. Log-normal distributional priors were selected to place 1.8 Mya at the median and shape priors encompassed late Pliocene to early Pleistocene, similar to Harding and Smith^94^. Fossil-calibrated divergence estimates separate two pairs of stoat clades (Beringia-NPC and East–West) at 4.47 Mya (±1.21 My, Pliocene), with the Beringia-NPC split dated within the early Pleistocene at 2.1 Mya (±0.6 My), over 1 million years before East–West cladogenesis (1 Mya ± 0.32; Supplementary Fig. 1). Phylogenies were visualized in FigTree v1.2.2 (http://tree.bio.ed.ac.uk/software/figtree/).

Traditional phylogenetic approaches were contrasted against TreeMix ancestral graphs modeling historic gene flow (migration events) among populations^95^. To accommodate genetic exchange between populations of the same species, TreeMix uses genome-wide allele frequency data to infer historical relationships among populations to maximize the composite likelihood of the sample covariance matrix (*W*_hat_), while modeling both cladogenesis and migration. TreeMix iterates over a standard bifurcating topology to identify a series of migration edges that most increase the likelihood score. TreeMix was run without default sample size corrections (-noss), because multiple populations were comprised of a single individual. We limited the number of migration events (-m) to three, to examine only the most influential events among the few groups examined. TreeMix simulations with *M. putorius* as the outgroup identified ancient geneflow between Beringia stoat ancestors and *M. putorius*. Simulations were re-run with *M. americana* as an outgroup to assess whether long-branch attraction between distantly diverged *M. erminea* and *M. americana* (>11Mya^96^) impacts migration inference in TreeMix, but results were consistent, regardless of the outgroup used. After TreeMix identified gene flow between *M. erminea* and *M. putorius* (Supplementary Fig. 4), we only show Admixture Graph results with *M. americana* as outgroup.

PCAs were generated in SNPrelate^97^. Diversity statistics (relatedness2, Supplementary Fig. 2; nucleotide diversity, Supplementary Table 11; *F*_ST_, Supplementary Table 2) were calculated using VCFtools. For ADMIXTURE^33^ analysis, sites with >80% missing data were removed and the lowest cross-validation (cv = 10- and 5-fold) score identified the most appropriate number of populations (K = 6) by iteratively leaving a sample out and reexamining the partitioning of genetic structure among the remaining samples.

### Introgression analyses

*F*-statistics were run in AdmixTools^41^ and MixMapper^51^ using *M. americana* as an outgroup after TreeMix identified ancient gene flow between *M. erminea* and *M. putorius* (Supplementary Fig. 4). *f2* statistics [*f*_*2*_(Pop_1_, Pop_2_)] quantify drift through summary statistics (e.g., allele frequencies, heterozygosity, covariance, and the probability of two lineages coalescing), with larger values indicating greater divergence^42^ (Supplementary Table 4). *f2* statistics were generated from the compute_moment_stats and compute_most_additive_trees functions in MixMapper with 1000 bootstraps and SNP blocks of 50 (1 per 50 bp). *f3-*statistics explicitly test for admixture (AdmixTools, 3PopTest) and considered all permutations where Source_1_, Source_2_, and the Target samples came from different populations. Populations (e.g., clades) were defined by phylogenetic relationships (Fig. 1) and ADMIXTURE results (Fig. 2c). A positive *f3* value does not necessarily indicate the absence of admixture^42^. Small sample sizes and the absence of a linkage map for *M. erminea* prevents linkage disequilibrium-based estimates of *N*_e_ and refined dating of admixture events.

To parse the backcrossing history of each hybrid sample, *f4-*statistics were used. We used block-jackknifing to accommodate non-independence between loci^42^. Although *f*-statistics alone cannot deduce the direction of gene flow in a system, admixture graph fitting can test whether a proposed evolutionary model fits the data well^45,51^. Admixture Graph^46^ fit hybrid individuals into a non-admixed tree topology (Supplementary Fig. 5a) based on *f4* results (Supplementary Tables 5 and 6). We ran an additional Admixture Graph simulation on the optimal (lowest minimal error) 4-taxa non-admixed backbone topology identified by Admixture Graph (Supplementary Fig. 5b; Supplementary Table 12) and again for Alaska-Yukon hybrids onto a 5-taxon backbone (Supplementary Fig. 5c) to evaluate how inferred relationships changed (Supplementary Fig. 6). We compared individuals from each population against members of alternative populations (e.g., *f*_*4*_ (Outgroup, NPC; East, Beringia)). We also compared hybrid individuals (as identified by *f3*-statistics) against individuals from ‘pure’ populations (e.g., *f*_*4*_(Outgroup, Hybrid; East, Beringia)) and admixed populations (e.g., *f*_*4*_ (East, SYT; Beringia; YTAK)) to decipher the backcrossing histories of hybrid samples and characterize patterns of gene flow across populations. *f4*-statistics are negative (*Z*-score ≤−5) if there is more allelic overlap between X and Y than between X and Z since the evolutionary split between Y and Z, and positive (*Z*-score ≥5) if there has been more recent gene flow between X and Z than between X and Y. *f4* results are in Table 2, Supplementary Tables 5 and 6, and Supplementary Fig. 7. Dawson et al.’s^12^ *N*_e_ Model 2 using multi-locus Approximate Bayesian Computation approximates mean clade *N*_e_ at: 250k for Beringia, 50k for NPC, 125k for East, and 75k for West; therefore, the combined *N*_e_ for NPC, East, and Beringia populations is 375k. *D* is tightly correlated with *N*_e_ and additional efforts to refine historical population sizes (Pleistocene, Pliocene) should improve the resolution of our admixture date estimates. Generational output was converted to years using a *M. erminea* generation time of 2 years^56^.

### Historical demography

PSMC^58^ was used on consensus genomic sequence data (using both complete and down-sampled sequence data) to characterize historical demography (Fig. 3d, e) by examining heterozygosity densities in a 100 bp sliding windows across the genome. PSMC was run twice for each individual, once utilizing all mapped sequence data (coverage 10–61×) and again on data down-sampled to 10× coverage (matching the lowest coverage sample) using the DownsampleSam tool (Picard). Results were scaled by a general mammalian mutation rate (2.2 × 10^−9^ per base pair per year^98^) and stoat generation time, resulting in distributions of *N*_e_ through time (Fig. 3d, e). One hundred PSMC bootstrap replicates were performed and plotted for both full-coverage and down-sampled data to confirm consistent distributional shapes and enable comparison across individuals, as PSMC is sensitive to variation in coverage depth (ideal coverage 18× (ref. ^99^)).

### Code availability

Our custom python spacing script is available on GitHub at https://github.com/jpcolella/Ermine_WGS_2018.

### Data Availability

Genomic reads are available in NCBI’s Sequence Read Archive, accession: SRP138989. Assembled FASTA files are available on Dryad (10.5061/dryad.bv2g720).

## Electronic supplementary material

Supplementary Information
