# Supplementary material for: Whole-genome analysis of Mustela erminea finds that pulsed hybridization impacts evolution at high latitudes
Source: Commun Biol. 2018 May 31;1:51. doi: 10.1038/s42003-018-0058-y (PMC6123727; doi:10.1038/s42003-018-0058-y)
Supplement: Supplementary file 1 — Supplementary Information [file 42003_2018_58_MOESM1_ESM.pdf]

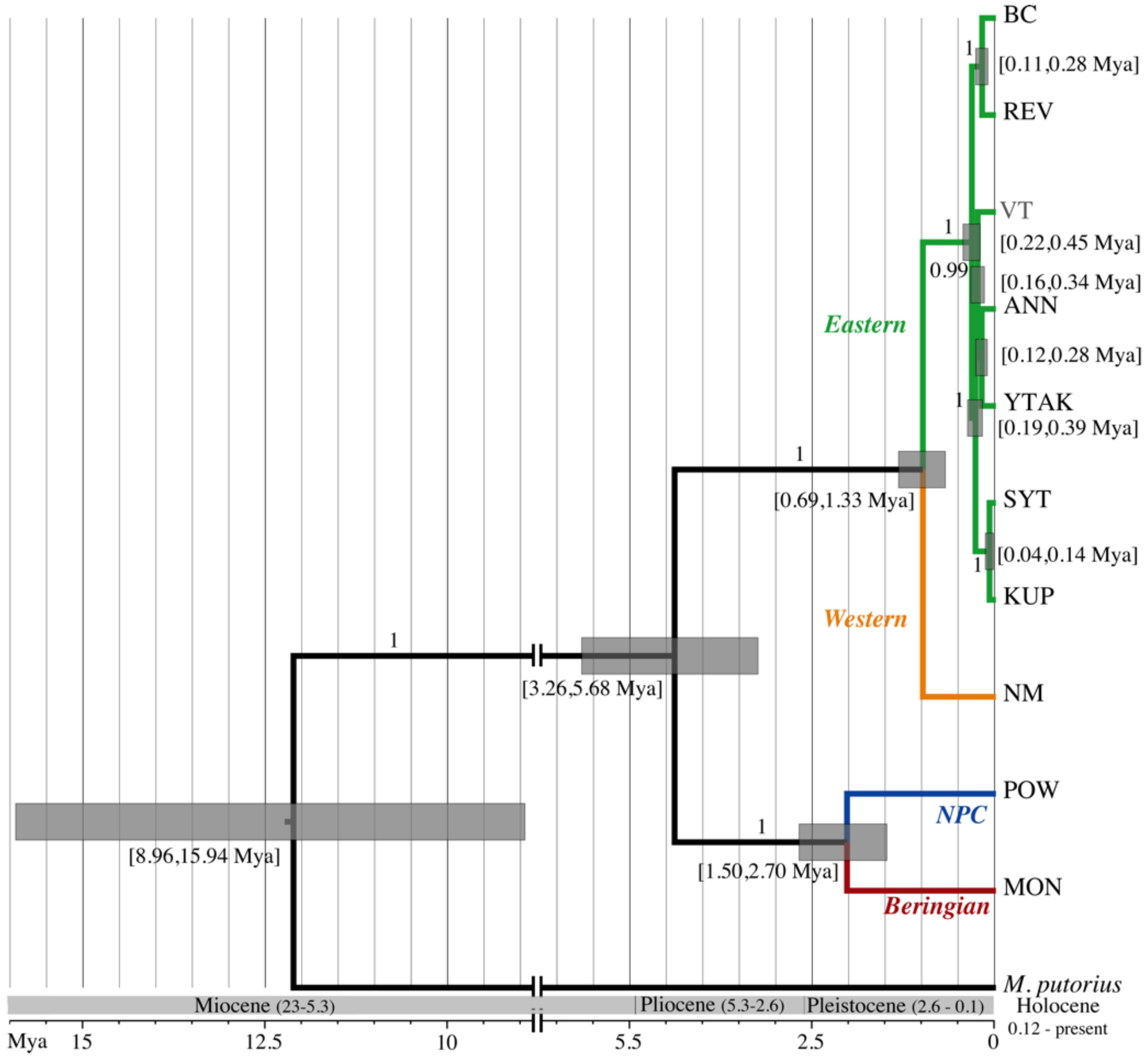

Supplementary Figure 1. Fossil calibrated (oldest *M. erminea* fossil: 1.8My[1]) mitochondrial genome tree showing divergence time estimates for each clade. Note ancient divergence between NPC (blue) and Beringia (red) clades relative to East-West cladogenesis.

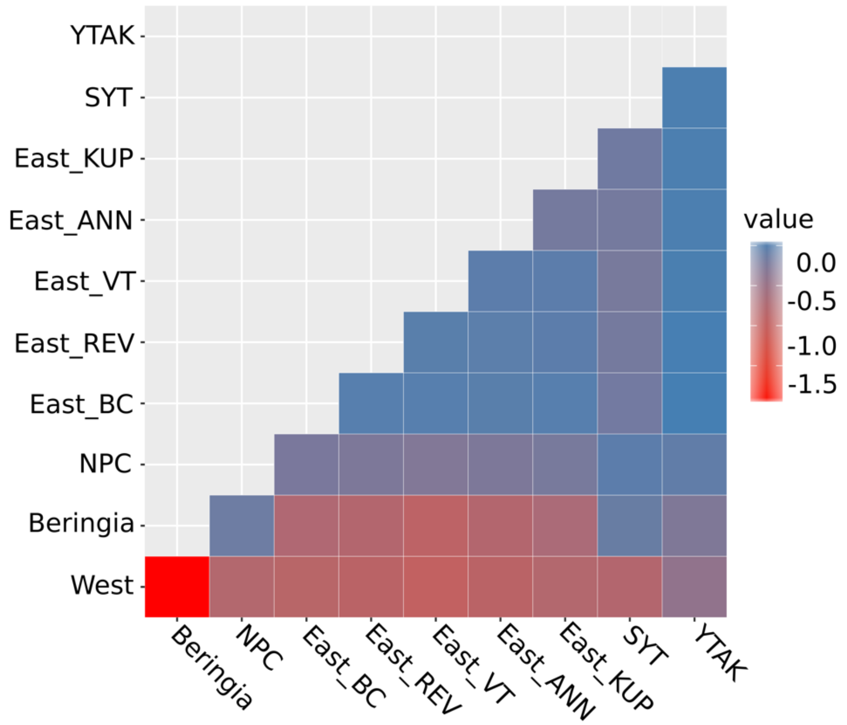

Supplementary Figure 2. Pairwise relatedness2 indices (proxy for F_{ST}^{2}) across all stoat samples with red indicating greater differentiation and blue, greater similarity between samples. These data first demonstrate substantial divergence of the West clade from all other stoats, especially from Beringia. Second, Beringia demonstrates greatest genetic similarly to its geographically isolated sister: the NPC clade. Lastly, both island and mainland hybrids are highly similar, supporting their origination from the same source populations despite temporal and geographic separation.

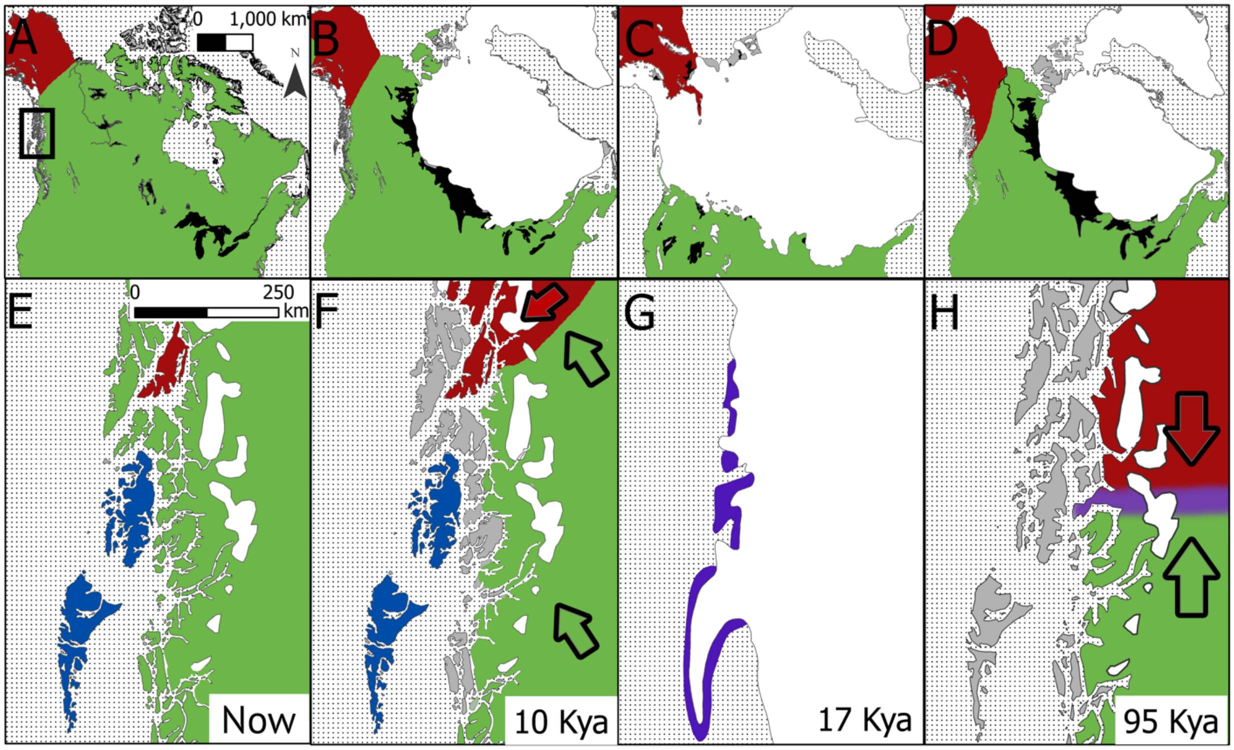

Supplementary Figure 3. Hypothesized climatically-driven isolation and secondary contact between stoat lineages. Eight, vertically paired panels with the greater extent North America in top panels (A-D) and lesser extent, North Pacific Coast (NPC) below (E-H; extent indicator included in panel A only) that reflect glaciation at four distinct time periods: Present (A, E), 10 Kya (B, F), 17 Kya (C, G), and 95 Kya (D, H; this period reflects similar glacial patterning to 10 Kya)[3]. Glacial ice is shown in white, lakes in black, and oceans with dotted shading. (A) Contemporary clade distributions and contact between East (green) and Beringia (red) lineages along the Alaska-Yukon Territory border; (B) Beringia-East contact zone historically extended farther south, enabling Beringian colonization of Admiralty Island along the NPC; (C) Refugial isolation during the last glacial period that maintained Beringia-East divergence; (D) Hypothesized ancient mainland hybridization event between Beringia and East lineages. Panels A-D demonstrate climatically-driven secondary contact and isolation between lineages isolated

in Beringia (Northwest) and East (eastern continental North America) refugia. Panels E-F demonstrate an alternative outcome of hybridization played out along North America's NPC, where a hybrid population (H) was isolated in a glacial refugium (G) and remained isolated as deglaciation and rising sea levels trapped the hybrid population on islands (F), resulting in further divergence and potentially speciation (E).

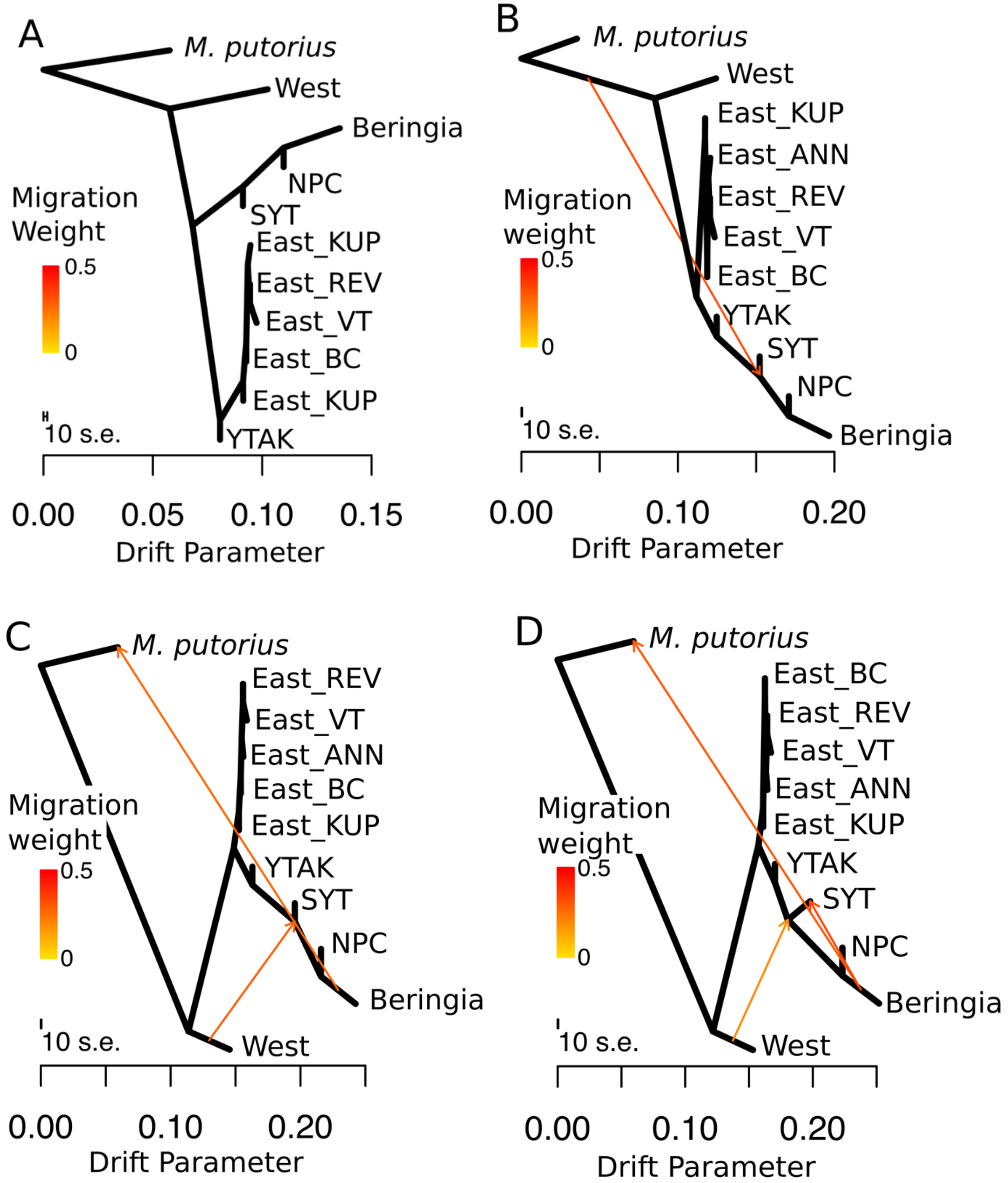

Supplementary Figure 4. TreeMix results for 0-3 migration events with *M. putorius* as an outgroup. (A) The TreeMix phylogeny without migration is consistent with our RAxML results (Fig. 1d); (B) The most likely migration event is between Beringia stoats and an unsampled ancestor of all *M. erminea*, consistent with New World colonization from Beringia and subsequent diversification in North America. Further, adding a migration event pulls both

contemporary hybrids (SYT, YTAK) towards their Beringia ancestor; (C) The second most supported migration event links West and SYT, supporting the hybrid ancestry of this individual; (D) and the third migration event links Beringia and SYT, highlighting the biased introgression (*f4*) between these samples.

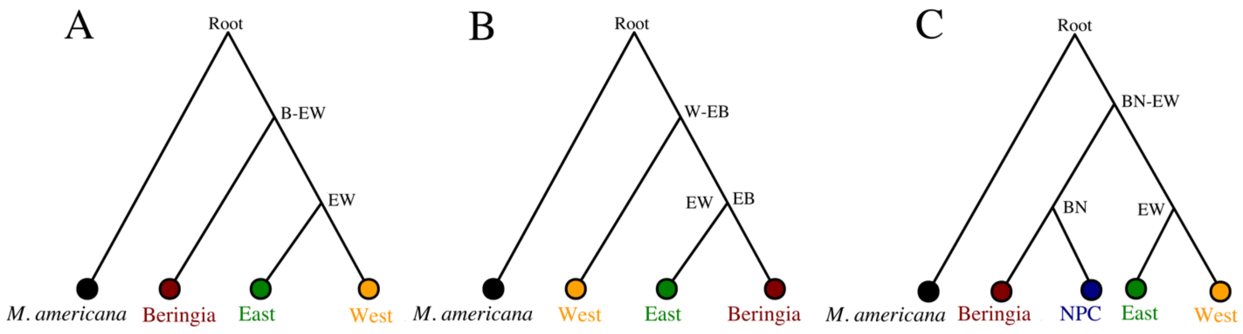

Supplementary Figure 5. Backbone topologies used to fit hybrid samples in AdmixtureGraph. (A) Represents the maximum-likelihood 4-taxa topology (e.g., RAxML, Fig. 1d); (B) the optimal (minimal error) 4-taxa backbone identified by AdmixtureGraph (Supplementary Table 12). When NPC is removed (from AdmixtureGraph and RAxML) West is consistently pulled towards the Root; and (C) the optimal 5-taxa backbone delimited in AdmixtureGraph, consistent with our RAxML results. Four and 5-taxon backbone phylogenies exclude and include NPC as a ‘pure’ population, respectively, to enable fitting of all three hybrid samples (A, B) or mainland samples only (C).

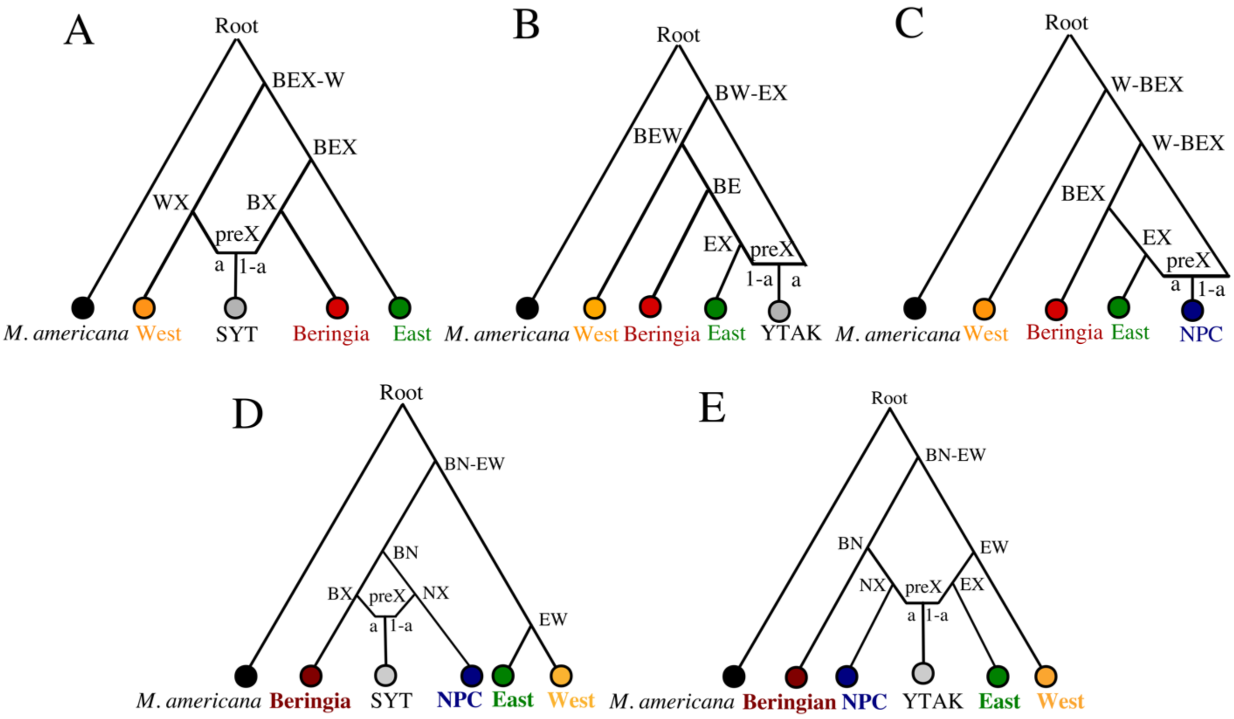

Supplementary Figure 6. Admixture graph fitting of each hybrid sample onto the lowest minimal error topology (Supplementary Table 7) 4- or 5-taxa non-admixed topology. Note that when NPC is removed from the backbone topology, AdmixtureGraph optimization pulls the West clade towards the root, however, in a 5-taxa backbone, relationships match our RAxML (Fig. 1d) and species trees (Supplementary Fig. 1). (A) Under this 4-taxa model, SYT is admixed between West and Beringia lineages; (B) YTAK is admixed between East and an ancestor of all *M. erminea*, consistent with the simulations based on the RAxML topology; and (C) NPC is admixed between East and a Beringia-East ancestor. Under a 5-taxa backbone phylogeny, including NPC as a ‘pure’ population: (D) shows SYT as the result of NPC-Beringia admixture. Since we know the NPC island clade is geographically separated from all others, this likely demonstrates the similarity between the two hybrid samples and biased introgression from Beringia; and (E) shows YTAK as the result of NPC-East admixture, again supporting biased introgression from East into YTAK and the shared hybrid history of YTAK and NPC.

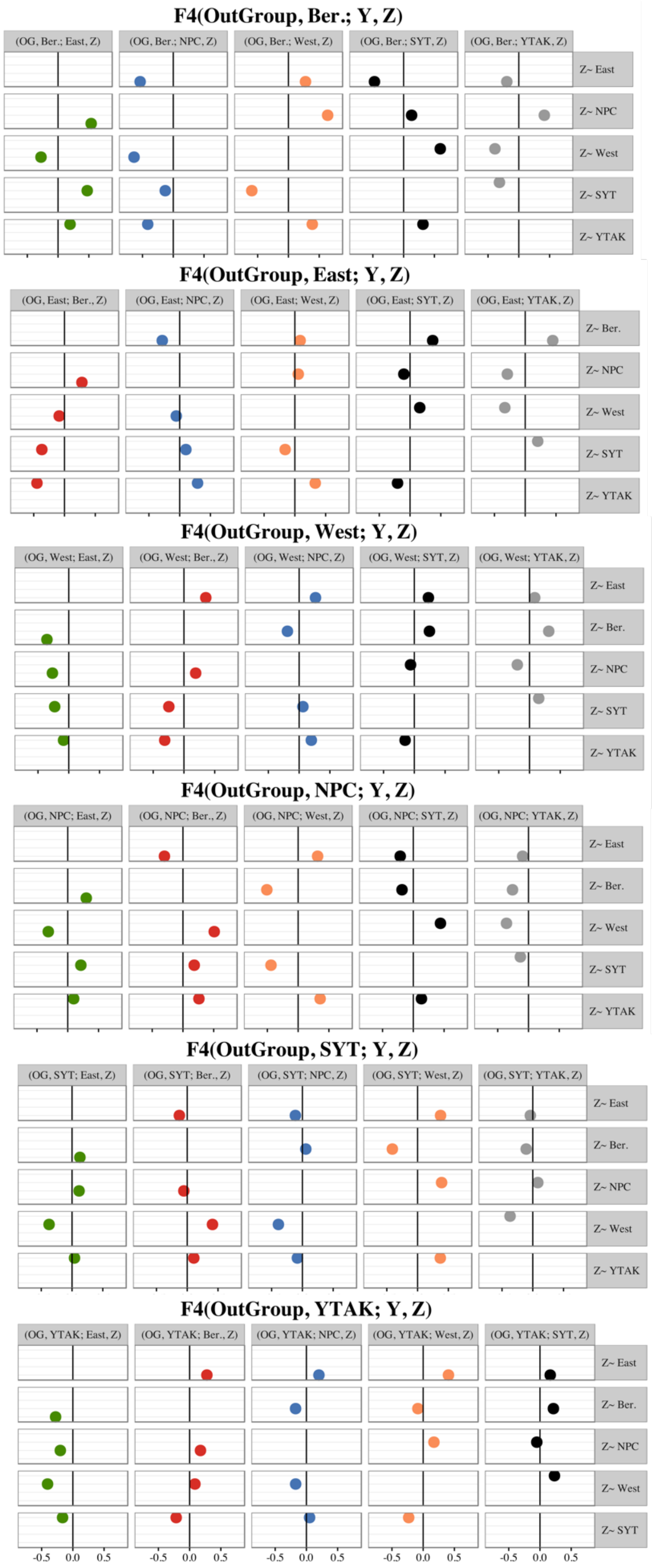

F4(OutGroup, Ber.; Y, Z)
(OG, Ber.; East, Z)
(OG, Ber.; NPC, Z)
(OG, Ber.; West, Z)
(OG, Ber.; SYT, Z)
(OG, Ber.; YTAK, Z)
Z~ East
Z~ NPC
Z~ West
Z~ SYT
Z~ YTAK
F4(OutGroup, East; Y, Z)
(OG, East; Ber., Z)
(OG, East; NPC, Z)
(OG, East; West, Z)
(OG, East; SYT, Z)
(OG, East; YTAK, Z)
Z~ Ber.
Z~ NPC
Z~ West
Z~ SYT
Z~ YTAK
F4(OutGroup, West; Y, Z)
(OG, West; East, Z)
(OG, West; Ber., Z)
(OG, West; NPC, Z)
(OG, West; SYT, Z)
(OG, West; YTAK, Z)
Z~ East
Z~ Ber.
Z~ NPC
Z~ SYT
Z~ YTAK
F4(OutGroup, NPC; Y, Z)
(OG, NPC; East, Z)
(OG, NPC; Ber., Z)
(OG, NPC; West, Z)
(OG, NPC; SYT, Z)
(OG, NPC; YTAK, Z)
Z~ East
Z~ Ber.
Z~ West
Z~ SYT
Z~ YTAK
F4(OutGroup, SYT; Y, Z)
(OG, SYT; East, Z)
(OG, SYT; Ber., Z)
(OG, SYT; NPC, Z)
(OG, SYT; West, Z)
(OG, SYT; YTAK, Z)
Z~ East
Z~ Ber.
Z~ NPC
Z~ West
Z~ YTAK
F4(OutGroup, YTAK; Y, Z)
(OG, YTAK; East, Z)
(OG, YTAK; Ber., Z)
(OG, YTAK; NPC, Z)
(OG, YTAK; West, Z)
(OG, YTAK; SYT, Z)
Z~ East
Z~ Ber.
Z~ NPC
Z~ West
Z~ SYT
-0.5 0.0 0.5

Supplementary Figure 7. *f4*-statistics [f_4(Outgroup, X; Y, Z)] results for each X population where Y (horizontal axis) and Z (vertical axis) represent all possible combinations of other populations, with *M. americana* as an outgroup. A significantly negative *f4*-statistic (e.g., point left of center in each column) indicates that there is biased allelic overlap between populations X and Y, whereas significantly positive indicates more shared alleles between X and Z. Non-significance (e.g., point on the center line) indicates Y and Z share more alleles with each other than with X. Error bars 5 times the standard error (mostly invisible outside the width of the points) highlight the extreme significance of these results, underlining the substantial inter-clade divergence within *M. erminea*. Columns are colored to reflect the Y population being compared. X assignment by plot: (A) Beringia, (B) East, (C) NPC, (D) West, (E) SYT, (F) YTAK.

Supplementary Table 1. Pairwise percent difference

**Clade**		East	NPC	East	East	Hybrid	East	East	West	Hybrid	Ber.[b]	OG[c]
	**Coll. Loc.**	**BC**	**POW**	**REV**	**VT**	**SYT**	**ANN**	**KUP**	**NM**	**YTAK**	**MON**	**OG**
East	**BC**		4.14	1.19	1.29	1.25	1.31	1.57	1.83	1.40	4.11	9.01
NPC	**POW**	19.70		4.17	4.19	4.09	3.99	4.43	4.20	4.16	2.71	8.51
East	**REV**	14.06	19.74		1.29	1.25	1.32	1.57	1.81	1.40	4.15	9.01
East	**VT**	13.62	19.97	13.57		1.23	1.26	1.51	1.79	1.36	4.15	9.10
Hybrid	**SYT**	17.97	15.96	17.96	18.00		1.19	1.40	1.78	1.26	4.08	8.86
East	**ANN**	14.15	19.71	14.00	13.70	17.97		1.54	1.82	1.16	4.15	8.72
East	**KUP**	14.38	19.71	14.55	14.10	18.17	14.63		2.03	1.53	4.36	9.32
West	**NM**	16.65	19.50	16.52	16.70	18.36	16.64	16.86		1.92	4.14	9.12
Hybrid	**YTAK**	16.06	18.86	16.00	15.65	17.66	16.12	16.42	17.77		4.23	8.93
Ber.	**MON**	20.60	12.57	20.63	21.13	13.39	20.59	20.65	19.13	18.75		9.22
OG	**OG**	22.46	22.31	22.42	22.35	21.61	22.48	22.70	18.48	22.79	20.30	

[a] lower triangle: percent pairwise difference between >3 million autosomal SNPs; upper triangle: percent pairwise difference between mitochondrial genomes. Darker shades of grey indicate greater pairwise divergence.

[b] Ber. = Beringia

[c] OG = outgroup (*Mustela putorius*)

Supplementary Table 2. Wright's F_{ST}

Populations	Avg. # Variants	Weighted F_{ST}	Mean F_{ST}
East v. NPC	251.01	0.33	0.17
East v. West	534.60	0.35	0.13
East v. Beringia	542.18	0.37	0.18

[a] Values based on 100bp sliding window

[b] Mean F_{ST} values >0.1are considered moderate to high. Weighted F_{ST} values > 0.25 indicate substantial divergence between clades.

Supplementary Table 3. AdmixTools *f3*-statistics

Target	Source 1	Source 2	*f3*	Standard Err	Z-score	SNPs
NPC	***East***	***Beringia***	***-0.0496***	***0.0094***	***-5.277****	***1160372***
SYT	***East***	***Beringia***	***-0.1673***	***0.0034***	***-48.662****	***2588407***
SYT	YTAK	Beringia	-0.1198	0.0045	-26.616	1752087
SYT	Beringia	West	-0.1096	0.0040	-27.343	2031423
SYT	NPC	East	-0.0733	0.0065	-11.360	1504688
SYT	NPC	YTAK	-0.0548	0.0061	-8.925	999537
SYT	West	NPC	-0.0534	0.0056	-9.459	1101322
YTAK	***East***	***Beringia***	***-0.1451***	***0.0031***	***-47.408****	***2545866***
YTAK	East	NPC	-0.1268	0.0026	-48.259	1311542
YTAK	SYT	East	-0.1096	0.0025	-43.499	2841843
YTAK	West	East	-0.0748	0.0024	-30.933	2664291
YTAK	West	SYT	-0.0744	0.0032	-23.342	2124843
YTAK	NPC	West	-0.0723	0.0036	-20.119	999138
YTAK	West	Beringia	-0.0640	0.0043	-15.032	1991741

*Significant Z-score (>5)

Supplementary Table 4. ***f2*-statistics population divergence**

	**East**	**NPC**	**SYT**	**West**	**YTAK**	**Beringia**
**East**	0.0000	0.0650	0.0284	0.0748	-0.0088	0.1015
**NPC**	0.0650	0.0000	0.0129	0.1100	0.0267	0.0230
**SYT**	0.0284	0.0129	0.0000	0.0798	-0.0041	0.0151
**West**	0.0748	0.1100	0.0798	0.0000	0.0569	0.1334
**YTAK**	-0.0088	0.0267	-0.0041	0.0569	0.0000	0.0529
**Beringia**	0.1015	0.0230	0.0151	0.1334	0.0529	0.0000

Supplementary Table 5. ***f4*****-statistics within** ***M. erminea***

W	*X*	*Y*	*Z*	*f4*	Z-score*
East	West	NPC	Ber.	0.15	22.80
East	YTAK	West	Ber.	0.22	23.04
East	West	SYT	Ber.	0.21	32.03
Ber.	East	SYT	West	0.38	32.56
East	West	YTAK	Ber.	0.24	42.36
Ber.	East	NPC	West	0.50	61.83
East	SYT	West	Ber.	0.55	64.65
East	NPC	West	Ber.	0.61	87.78

* All Z-scores are significant (> 5).

Supplementary Table 6. *f4*-statistic tests with *M. americana* outgroup (OG)

W	X	Y	Z	*f4*	Z-scores*
OG	Beringia	East	NPC	0.5381	88.851
OG	Beringia	East	SYT	0.4736	62.909
OG	Beringia	East	YTAK	0.1951	23.007
OG	Beringia	West	East	0.2773	85.269
OG	Beringia	West	NPC	0.6372	100
OG	Beringia	West	SYT	0.5957	100
OG	Beringia	West	YTAK	0.3875	62.77
OG	Beringia	SYT	NPC	0.1299	9.769
OG	Beringia	YTAK	NPC	0.4147	48.393
OG	Beringia	YTAK	SYT	0.3147	27.843
OG	East	Beringia	NPC	0.2832	41.676
OG	East	Beringia	West	0.0861	19.748
OG	East	Beringia	SYT	0.3681	54.142
OG	East	Beringia	YTAK	0.4473	83.495
OG	East	NPC	SYT	0.1009	9.026
OG	East	NPC	YTAK	0.2907	41.461
OG	East	West	NPC	0.0553	10.812
OG	East	West	SYT	0.1578	22.393
OG	East	West	YTAK	0.3314	56.935
OG	East	SYT	YTAK	0.2031	23.241
OG	NPC	East	Beringia	0.3008	31.794
OG	NPC	East	SYT	0.2128	26.195
OG	NPC	East	YTAK	0.0957	14.846
OG	NPC	West	Beringia	0.5085	70.422
OG	NPC	West	East	0.3166	76.992
OG	NPC	West	SYT	0.441	70.758
OG	NPC	West	YTAK	0.3578	69.802
OG	NPC	SYT	Beringia	0.1835	22.851
OG	NPC	YTAK	Beringia	0.2598	28.847
OG	NPC	YTAK	SYT	0.1321	16.507
OG	West	Beringia	East	0.3548	100
OG	West	Beringia	NPC	0.1904	35.861
OG	West	Beringia	SYT	0.2455	50.398
OG	West	Beringia	YTAK	0.3128	84.295
OG	West	NPC	East	0.2659	58.322
OG	West	NPC	SYT	0.0614	7.772
OG	West	NPC	YTAK	0.1979	36.19
OG	West	SYT	East	0.2288	48.123

OG	West	SYT	YTAK	0.1499	24.874
OG	West	YTAK	East	0.0848	18.987
OG	SYT	East	Beringia	0.1278	9.286
OG	SYT	East	NPC	0.1143	9.849
OG	SYT	East	YTAK	0.0399	6.165
OG	SYT	NPC	Beringia	0.0549	5.66
OG	SYT	West	Beringia	0.4103	42.167
OG	SYT	West	East	0.3731	86.48
OG	SYT	West	NPC	0.3902	50.232
OG	SYT	West	YTAK	0.3692	79.363
OG	SYT	YTAK	Beringia	0.1069	8.162
OG	SYT	YTAK	NPC	0.0816	8.047
OG	YTAK	Beringia	East	0.2764	27.122
OG	YTAK	Beringia	NPC	0.1736	21.245
OG	YTAK	Beringia	SYT	0.215	23.691
OG	YTAK	NPC	East	0.2006	22.649
OG	YTAK	NPC	SYT	0.051	5.704
OG	YTAK	West	Beringia	0.0849	10.175
OG	YTAK	West	East	0.4048	90.67
OG	YTAK	West	NPC	0.1721	26.594
OG	YTAK	West	SYT	0.2321	34.026
OG	YTAK	SYT	East	0.1645	19.083

[a] All comparisons with only a single hybrid (NPC, SYT, or YTAK) included in the test and *M. americana* as an outgroup (OG).
*Z-scores are positive for ease of interpretation and all are significant (> 5). Rotating the order of a pair (e.g., YZ to ZY) switches the sign +/- of both the *f4*-statistic and the Z-score but does not change the interpretable relationships.

Supplementary Table 7. AdmixtureGraph minimal error estimates

(A)

	**Minimal Error**	
	5-taxa backbone	
**Nodal Codes[a]**	**SYT**	**YTAK**
B-N	***145116.00***	147595.50
B-E	148680.80	142512.30
B-W	149402.10	150093.00
N-E	145149.90	***139919.00***
N-W	146026.20	147804.70
E-W	151694.10	150582.00
NB-E	148680.80	142512.30
NB-W	149402.10	150093.00
WE-B	149402.10	150093.00
WE-N	146026.20	147804.70
NB-EW	149402.10	150093.00
WEB-NB	149402.10	150092.90
WEB-WE	152147.80	155840.20
WEB-B	149402.10	150092.90
WEB-N	146026.20	147692.00
WEB-E	151694.10	150575.70
WEB-W	152147.80	156049.50
EW-E	151694.10	149943.30
W-WE	152147.80	155840.20
N-NB	146026.20	147804.70
BN-B	149402.00	150093.00
B-EB	na	na
EB-E	na	na

(B)

	**Minimal Error**					
	AdmixtureGraph 4-taxa backbone			*RaxML 4-taxa backbone*		
**Nodal Codes[a]**	**SYT**	**YTAK**	**NPC**	**SYT**	**YTAK**	**NPC**
B-E	55132.96	47383.25	70165.58	***72727.22***	61506.26	***82798.40***
B-W	***52304.13***	47105.77	62996.35	73410.49	69668.84	83144.70
E-W	52309.13	46983.80	62816.24	74264.60	69046.22	82977.55
WE-B	na	na	na	73410.49	69.668.80	83144.46
WEB-B	52362.71	39848.05	63877.58	73792.47	67922.87	84830.71
WEB-E	52371.27	***39505.20***	63762.10	73685.00	***61351.88***	82865.19
WEB-W	58496.32	62257.46	67371.90	74368.69	70895.87	83219.90
EW-E	na	na	na	73685.00	64299.36	82865.19

W-WE	na	na	na	74368.69	71436.82	83215.46
B-EB	52632.71	47061.96	63877.58	na	na	na
EB-E	52371.27	46983.80	***62762.10***	na	na	na
EB-W	52309.13	47061.96	62996.35	na	na	na

[a] Hybrids can be descended from sister clades (connected by '-') or a group of related clades (each separated by '/'), represented by the clades' first letter (B = Beringia, N = NPC, E = East, W = West).
[b] ***bold italics***, indicates the most likely ancestral relationships for each hybrid sample
[c] Not all relationships are present in both topologies (Supplementary Fig. 5), these relationships are denoted with 'na'.

Supplementary Table 8. Range of hierarchical admixture date estimates

Admixed	Source 1	Source 2	reps	alpha	Mixed Drift	Years (Ne 300k)	Years (Ne 375k)	Years (Ne 450k)
SYT	Beringian	East	852	0.48-0.59	0.00-0.00	0	0	0
YTAK	East	Beringia	852	0.52-0.78	0.00-0.00	0	0	0
NPC	Beringia	East	865	0.48-0.73	0.00-0.23	0-315,197.2	0-393,996.5	0-472,795.8

Supplementary Table 9. Voucher specimen museum identification numbers and metadata

MSB#[a]	Locality	Abrv.[b]	Clade[c]	HiSeq lanes	Pairs Sequenced	Bases (Gb)	Coverage
152905	British Columbia	BC	East	4	996,730,113	199.35	61.16
221783	Prince of Wales, AK	POW	NPC	4	122,281,422	24.70	8.40
199855	Revillagigedo Is., AK	REV	East	4	413,868,733	83.60	27.03
43333	Vermont	VT	East	1	261,862,572	52.37	17.10
145234	Yukon Territory	SYT	X	1	216,684,577	43.77	15.42
148962	Annette Is., AK	ANN	East	1	242,559,925	49.00	15.70
149365	Kupreanof Is., AK	KUP	East	1	225,414,511	45.53	12.67
144524	New Mexico	NM	West	1	225,740,082	45.60	14.58
215111	Yukon-Alaska border	YTAK	X	1	158,943,442	32.11	10.29
248153	Mongolia	MON	Ber.	1	220,469,640	44.53	14.10

[a] Museum accession numbers associated with voucher specimens held at the Museum of Southwestern Biology (MSB).
[b] Abrv = abbreviation
[c] NPC = North Pacific Coast Island clade; X = Alaska-Canada hybrid population; Ber. = Beringia Clade

Supplementary Table 10. SNP filtering and quantity per analysis

Analysis	#SNPs	Filtering	Outgroup
**TreeMix (*Martes*)**	332,621	Missing data removed, pseudo-chromosomes, spaced	*Martes*
**TreeMix (*M. putorius*)**	3,419,651	Missing data removed, pseudo-chromosomes, spaced	*M. putorius*
**ADMIXTURE**	14,386,411	2nd round of filtering + Sites with >80% missing data removed	None
**PCA**	15,673,343	1st and 2nd rounds of filtering, pseudo-chromosomes	None
**F-statistics, MixMapper**	20,706,783	Spaced, pseudo-chromosomes	*M. putorius*
**Relatedness2**	41,300,628	1st round of filtering + MAF (0.99), pseudo-chromosomes, spaced	*Martes*
**F_{ST} and π**	58,719,680	1st round of filtering + MAF (0.99)	*Martes*
**2nd round of Filtering, RaxML**	59,074,574	1st round of filtering + MAF (0.01)	*Martes*
**1st round of Filtering**	165,789,498	Indels removed, minimum depth (2), minimum genotype quality (30), minimal mean depth (10), maximum MAF (0.99), Martes outgroup	*Martes*

Supplementary Table 11. Nucleotide diversity (π) based on 100bp sliding window averages

Lineage	Avg. # Variants	π
Beringia	556.65	0.0012
East	78.99	0.0019
NPC	100.02	0.0010
West	106.40	0.0008

Supplementary Table 12. AdmixtureGraph minimal error backbone topology identification

Topology	Minimal Error
4-taxa backbone Optimization	
(OG,(W,(E,B)))	***22,471.35***
(OG,(E,(W,B)))	35,321.57
(OG,(B,(E,W)))	26,845.98
5-taxa backbone Optimization	
(OG,(W,E),(N,B))	***83,218.04***
(OG,(W,E),(N,B))	99,425.96
(OG,(W,E),(N,B))	106,980.00

Supplementary References

1. King, C. M. *Mustela erminea*. *Mammalian Species* No. 195, 1–8 (1983).

2. Manichaikul, A., Mychaleckyj, J.C., Rich, S.S., Daly, K., Sale, M., Chen, W.-M. Robust relationship inference in genome-wide association studies. *Bioinformatics* **26**, 2867–2873 (2010).

3. Dyke, A. S., Moore, A. & Robertson, L. Deglaciation of North America. *Natural Resources Canada, Ottawa* Open File 1574 (2003).
